# Age-Period-Cohort Analysis of Chronic Obstructive Pulmonary Disease Mortality in Japan, 1950–2004

**DOI:** 10.2188/jea.JE20110092

**Published:** 2012-07-05

**Authors:** Truong-Minh Pham, Kotaro Ozasa, Tatsuhiko Kubo, Yoshihisa Fujino, Ritsu Sakata, Eric J. Grant, Shinya Matsuda, Takesumi Yoshimura

**Affiliations:** 1 Department of Epidemiology, Radiation Effects Research Foundation, Hiroshima, Japan; 2 Department of Preventive Medicine and Community Health, School of Medicine, University of Occupational and Environmental Health, Kitakyushu, Fukuoka, Japan; 3 Department of Food and Health Sciences, International College of Arts and Sciences, Fukuoka Women’s University, Fukuoka, Japan

**Keywords:** age-period-cohort model, birth cohort analysis, COPD, secular trends

## Abstract

**Background:**

We examined trends in chronic obstructive pulmonary disease (COPD) mortality in Japan.

**Methods:**

An age-period-cohort model was used to examine trends in COPD mortality by age, time period, and birth cohort among adults aged 40 years or older from 1950 to 2004.

**Results:**

During the study period, the age-standardized rate of COPD mortality substantially decreased from 71.3 per 100 000 to 19.7 in men and from 41.7 to 4.3 in women. The period effects rapidly declined during the early period in both sexes. They have increased in recent years in men but have continued to decrease in women. The cohort effects began increasing in the 1865–1869 birth cohort, peaked in the 1880–1889 cohort, and decreased thereafter among the recent cohorts.

**Conclusions:**

An early decrease in the period effects might have been associated with changes in disease structure and misclassification of COPD in the early period. Changes in cohort effects might have been mostly associated with changes in cigarette consumption and smoking prevalence in the Japanese population. Changes in those effects may also be a proxy for other social changes over time.

## INTRODUCTION

Chronic obstructive pulmonary disease (COPD) is an important cause of morbidity and mortality worldwide. According to the Global Burden of Disease Study, it was the sixth leading cause of death in the world and the fifth leading cause of death in developed countries.^1^^,^^2^ Furthermore, COPD prevalence and morbidity are expected to increase in coming decades as the proportion of elderly adults in the population increases.^2^ COPD was responsible for an estimated 2.75 million deaths worldwide in 2000, half of which were in the Western Pacific region.^3^ In Japan, COPD has ranked among the 10 leading causes of death since 2000.^4^ The prevalence of COPD was estimated to be 8.6% of all adults aged 40 years or older according to the Nippon COPD Epidemiology Study.^5^ Another study in Japan reported an incidence rate of 0.81 per 100 person-years in men and 0.31 in women among study participants aged 25 to 74 years.^6^

In the present study, we examined secular trends in COPD mortality, emphasizing the contributions of age at death, time period, and birth cohort to COPD mortality trends between 1950 and 2004 in Japan.

## METHODS

### Data sources

Sex- and age-specific mortality data on COPD were obtained from the Vital Statistics of Japan, 1950–2004.^7^ During that period, COPD subtypes were classified according to several versions of the International Classification of Diseases and Injuries (ICD), as shown in the Table. The classification followed a previous report that evaluated ICD code changes in selected European countries.^8^

**Table.** Description of COPD codes from ICD-6 to ICD-10

ICD Revisions (Calendar years)	6th and 7th (1950–1967)	8th (1968–1978)	9th (1979–1994)	10th (1995–2004)
Bronchitis, unqualified	501	490	490	J40, J42
Chronic bronchitis	502	491	491	J41
Bronchiectasis	526	518	494	J47
Pulmonary emphysema	527	492	492	J43
Other chronic airway obstructive	—	—	496	J44

### Data analysis

The analyzed data were restricted to deaths at age 40 or older, since COPD usually affects adults in that age group. We first estimated age-standardized rates (ASRs) per 100 000 persons, truncated for age 40 or older using the direct method according to the World Standard Population.^9^ The data were tabulated into 10 five-year age groups (from age 40 to 45 years to 85+ years), 11 five-year time periods (from the 1950–1954 year interval to the 2000–2004 year interval), and 20 five-year birth cohorts (from the 1865–1869 year interval to the 1960–1964 year interval). We analyzed the effects of age, time period, and birth cohort on COPD mortality using an age-period-cohort model (APC model) by means of Poisson regression. The full APC model is given by:

$$\log({\text{d}}_{ij}/{\text{p}}_{ij})=\text{intercept}+{\text{age}}_{i}+{\text{period}}_{j}+{\text{cohort}}_{k}$$

where cohort = period − age; d*_ij_* denotes the number of deaths in the *i*th age group, *j*th time period, and *k*th birth cohort; p*_ij_* denotes the respective population at risk; age*_i_* denotes the effect of the *i*th age group for *i* = 1,…, *A*; period*_j_* denotes the effect of the *j*th time period for *j* = 1,…, *P*; and cohort*_k_* denotes the effect of the *k*th birth cohort for *k* = 1,…, *C* = *A* + *P* − 1, with *k* = *A* − *i* + *j*. These estimated parameters were centered to satisfy the following:

$$\sum_{i}{\text{age}}_{i}=\sum_{j}{\text{period}}_{j}=\sum_{k}{\text{cohort}}_{k}=0$$

In the full 3-factor model, there is an inherent identification problem that is originally induced by the exact linear relationship among the 3 particular variables.^10^^,^^11^ A number of methods have been proposed to solve this. In the present analysis, we applied the intrinsic estimator method to address the identification problem and to provide parameter estimates.^12^^–^^14^ Figures were drawn with SigmaPlot version 11.0 software. Statistical analyses were performed using Stata version 9.2 software.

## RESULTS

**Figure 1.**
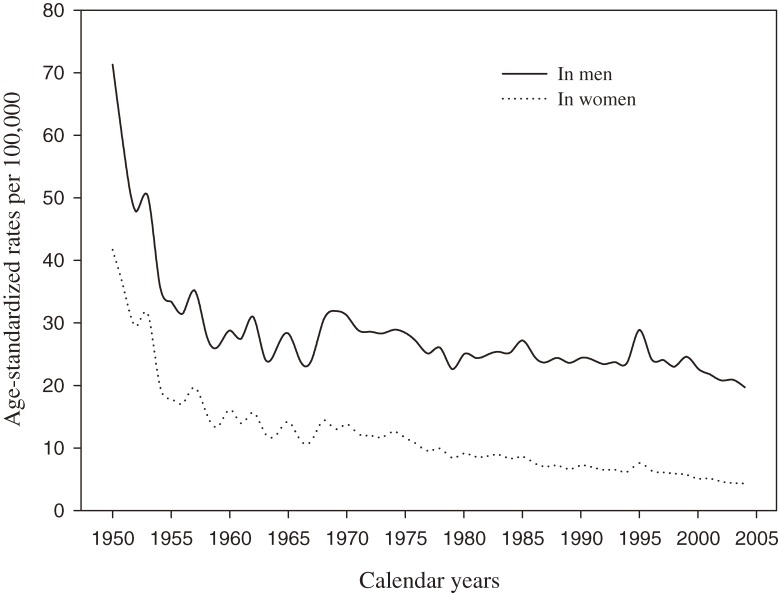
Age-standardized rate of chronic obstructive pulmonary disease mortality per 100 000 persons between 1950 and 2004

**Figure 2.**
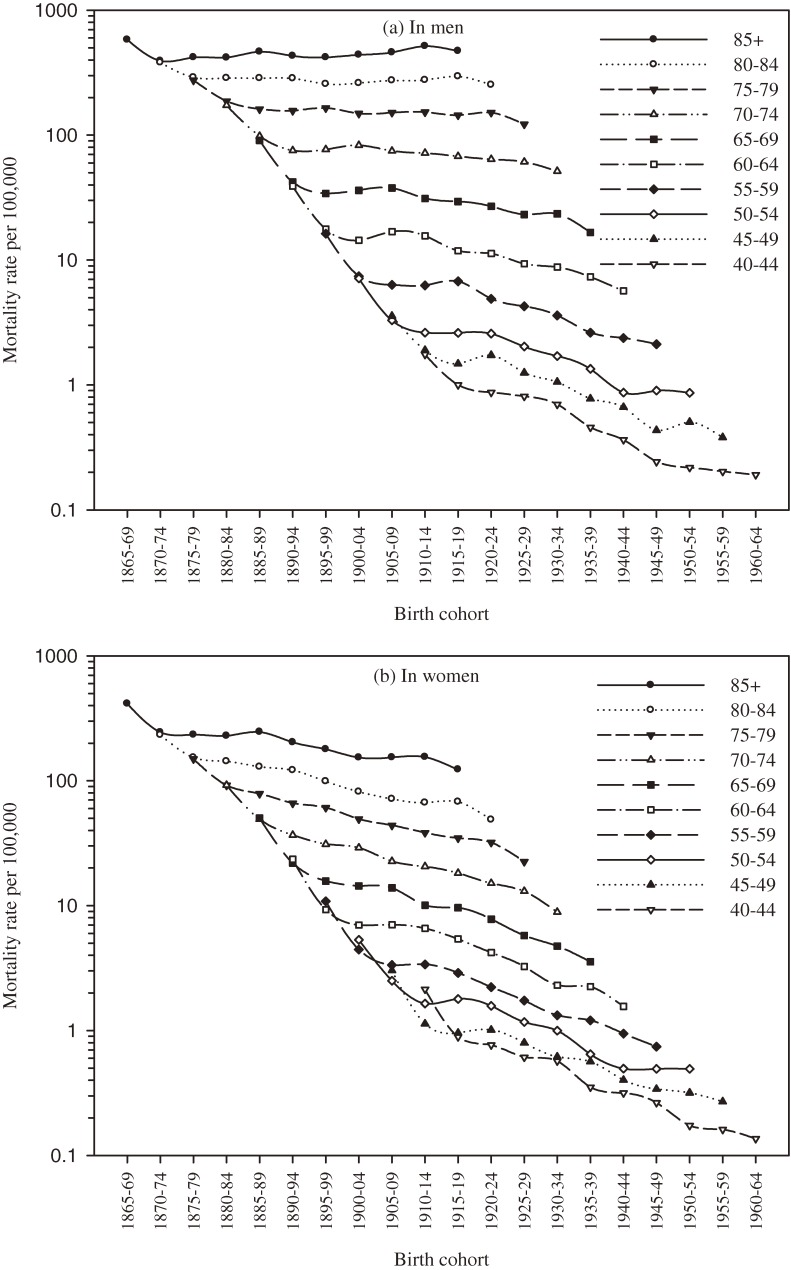
Age-specific rate of chronic obstructive pulmonary disease mortality per 100 000 persons by birth cohort

From 1950 to 2004, the truncated ASRs of COPD mortality substantially decreased, from 71.3 per 100 000 to 19.7 in men and from 41.7 to 4.3 in women (Figure 1). The greatest decreases were observed from 1950 to 1956, when ASRs decreased by half, to 31.5 in men and 17.1 in women. The downward trends then slowed and fluctuated: in 1967, the ASR decreased to 24.1 in men and to 11.2 in women. In 1968, the ASRs increased to 30.4 in men and 14.4 in women, then slowly decreased until 2004. Figure 2 shows that death rates increased with age in all birth cohorts, especially among adults 65 years or older. In most age categories, death rates were higher in older birth cohorts than in more recent birth cohorts.

**Figure 3.**
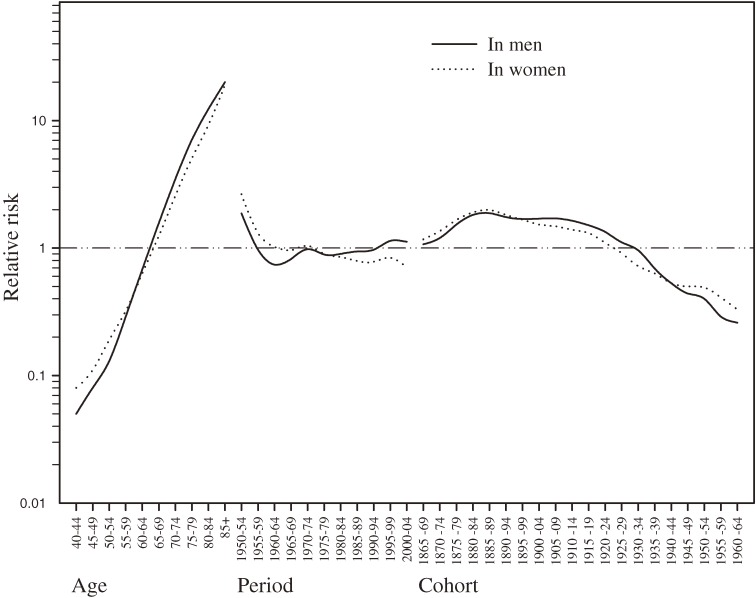
Effects of age, time period, and birth cohort on chronic obstructive pulmonary disease in men and women

The effects of age, time period, and birth cohort were expressed in terms of relative risk and are depicted graphically in Figure 3. The age effects increased with age in both sexes. The period effects rapidly declined in both sexes during the first 10-year period, ie, from 1950, and started increasing in the 1960s. In men, period effects tended to increase in recent years, while they continued to decrease in women. With regard to birth cohort, we observed increased effects in both sexes, starting from the 1865–1869 cohort. The highest values were observed in the 1880–1889 cohorts, and they decreased continuously thereafter up to the last birth cohort of 1960–1964.

## DISCUSSION

COPD is a group of chronic progressive lung diseases characterized by airflow limitations that are not fully reversible.^15^ Tobacco smoking is an important risk factor for this condition.^16^^,^^17^ Other risk factors include air pollution, exposure to occupational dust or chemical materials, and genetic predisposition associated with apha-1 antitrypsin deficiency.^16^ COPD is associated with a substantial disease burden worldwide, which is projected to double within the next 2 decades.^2^ In Japan, awareness of the condition is negligible as compared with other chronic conditions such as cancer and circulatory diseases. There have been only a few studies of Japanese with the condition. The Nippon COPD Epidemiology Study found a higher prevalence of airflow limitation in ever-smokers than in non-smokers among 2343 adults aged 40 years or older with valid spirometry data.^5^ Other studies in Japan also reported that smoking increased the risk of COPD.^6^^,^^17^^,^^18^

**Figure 4.**
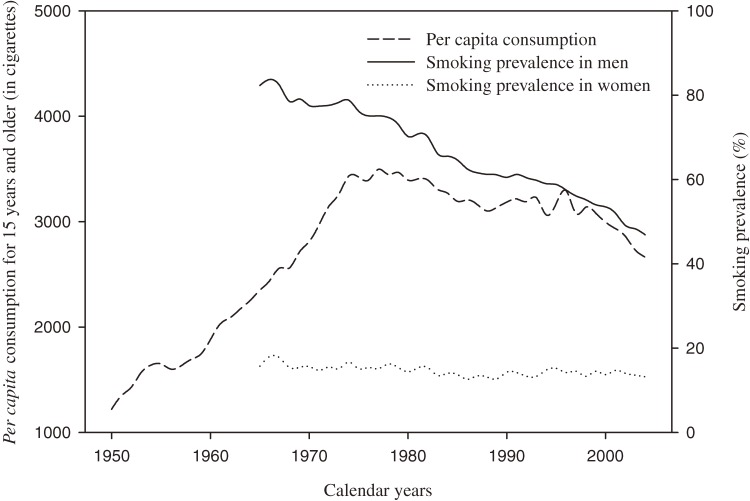
Trends in per capita consumption of cigarettes (1950–2004) and smoking prevalence (1965–2004) in Japan (Source: *Tobacco or Health*, Japan Health Promotion and Fitness Foundation; http://www.health-net.or.jp/tobacco/menu02.html)^19^

Cigarette consumption and smoking prevalence markedly increased after World War II. The trends in per capita consumption of cigarettes (1950–2004) and smoking prevalence rates (1965–2004) in Japan are shown in Figure 4, using data from *Tobacco or Health*, published by the Japan Health Promotion and Fitness Foundation.^19^ Tobacco consumption per capita (age ≥15 years) began to increase at the end of World War II and peaked in the 1970s. Consumption then fluctuated and decreased slowly until the end of the study period. Smoking prevalence rates peaked before or around 1965, when the prevalence of cigarette smoking at age 20 years or older was about 82% in men and 16% in women.^19^^,^^20^ After that, prevalence rates have decreased every year, falling to 60% and 14% in men and women, respectively, in 1990, and to 47% and 13%, respectively, in 2004.^19^ However, prevalence rates in young women have increased recently (about 16% of women <30 years smoke currently vs 6% in 1965).^19^ Furthermore, smoking prevalence rates tended to be lower in recent cohorts than in older cohorts.^19^ Hence, marked changes in tobacco consumption and smoking prevalence may have influenced changes in period and cohort effects on COPD mortality rates. For instance, the increase in tobacco consumption until the 1970s might correspond to the early increase in cohort effects. The subsequent decrease in smoking prevalence might have resulted in a decreased risk of COPD among recent cohorts, but some slowing in the decrease in women may reflect increased smoking prevalence among young women.^19^ In addition, the sex difference in smoking prevalence in the past (very high in men, but relatively low in women), as well as the difference in the decrease in rates between sexes over time, may explain recent differences between sexes in both period and cohort effects. To hasten reductions in COPD and other smoking-related deaths, population-based tobacco-free programs should be further implemented and promoted.

Other factors, such as changes in the age structure of populations, lifestyle, health care, and changes in ICD version, might have influenced COPD trends. The Japanese population increased from 83 million people in 1950 to 127 million in 2004. The proportion of people aged 65 or older increased from 5% in 1950 to 7% in 1970 and to 20% in 2004.^21^ The ASRs of COPD generally decreased during the overall study period, but with some fluctuation. The high rate and subsequent rapid decline in the early 1950s may have been due to the predominance of infectious diseases, the misclassification of acute or poorly understood disease entities as COPD, and subsequent improvement in those situations. The high mortality attributed to COPD among young age groups during that period is possible evidence of misclassification, and the drastic decrease in period effects during the earliest time period may have been due the changes in disease structure and medical care in Japan during the period. An increase in 1968 may have been due to the change from ICD-7 to ICD-8. However, it is unclear how the changes in ICD version might have affected the increase or how an increase in ASRs might have affected trends in period and cohort effects, although some increase in period effect occurred during 1965–1969. In addition, improvements in the availability of health care may have had an important influence on time period and birth cohort effects. The decline in recent birth cohorts may be due to healthier lifestyles or health care improvement. Thus, period and cohort effects may be proxies for secular changes in several factors such as smoking and available health care.

The APC model can consider all 3 factors—age, time period, and birth cohort—as potential variables of interest, although cohort effects are not usually considered in analyses of trend in the mortality and morbidity of specific conditions. The inherent challenge when applying the full APC model is the identification problem, which is induced by the exact linear dependency among the 3 variables, that is cohort*_A_*_−_*_i_*_+_*_j_* = period*_j_* − age*_i_*. Although there have been a number of proposed approaches for this problem,^10^^,^^11^ there is no consensus regarding the optimal method. Thus, comparison of estimates derived from different methods should be carefully interpreted.

Several limitations of the present study should be mentioned. First, treatments and understanding of the ICD codes for COPD by medical physicians may not have been consistent between the different ICD versions. Practical diagnostic classification for COPD may have differed between the periods, and these differences might have influenced the trends. Second, we could not adjust for other potential confounding factors, due to the unavailability of related information in mortality data from the Vital Statistics.

In summary, an early decrease in COPD period effects might have been associated with changes in disease structure in Japan and misclassification of COPD during the early period. Changes in cohort effects might have been mostly associated with changes in cigarette consumption and smoking prevalence in the Japanese population. The changes in those effects may also be a proxy for many underlying secular changes in Japan, including changes in diagnostic criteria, demographic factors, and advances in medical practice and health care.
